# Long-term remission of a Her2/neu positive primary breast cancer under double monoclonal antibody therapy with trastuzumab and bevacizumab

**DOI:** 10.2478/raon-2013-0083

**Published:** 2014-04-25

**Authors:** Robert Königsberg, Julia Maierhofer, Tanja Steininger, Gabriele Kienzer, Christian Dittrich

**Affiliations:** 1 Ludwig Boltzmann Institute for Applied Cancer Research (LBI-ACR VIEnna) – LB Cluster Translational Oncology, 3^rd^ Medical Department – Centre for Oncology and Haematology, Kaiser Franz Josef-Spital, Vienna, Austria; 2 Applied Cancer Research – Institution for Translational Research Vienna (ACR –ITR VIEnna), Vienna, Austria; 3 Institute for Radiodiagnostics, Kaiser Franz Josef-Spital, Vienna

**Keywords:** breast cancer, Her2/neu, trastuzumab, bevacizumab, VEGF

## Abstract

**Background:**

The attempt to act on several signalling pathways involved in tumour development simultaneously appears to be more attractive than attacking a single target structure alone. Vascular endothelial growth factor (VEGF) over-expression is frequently observed in human epidermal growth factor receptor 2 (Her2/neu) positive patients with breast cancer and over-expression of the proto-oncogene Her2/neu is associated with an up-regulation of VEGF.

**Case report:**

The case of a Her2/neu positive patient with breast cancer who refused cytotoxic chemotherapy with its potential side effects as well as mastectomy is presented. Our patient has been receiving the combined double administration of bevacizumab and trastuzumab for more than 4 years.

**Conclusions:**

This case report shows that (a) the combined double administration of bevacizumab and trastuzumab was be clinically effective. (b) The combination of bevacizumab and trastuzumab is safe and non-toxic. (c) Bevacizumab and trastuzumab can be used as a long-term application.

## Introduction

Vascular endothelial growth factor (VEGF) over-expression is frequently observed in human epidermal growth factor receptor 2 (Her2/neu) positive patients with breast cancer. Over-expression of the proto-oncogene Her2/neu is associated with an up-regulation of VEGF. There is, therefore, a biological rationale for targeting both Her2/neu and VEGF pathways in patients with Her2/neu positive breast cancer. We present the case of a postmenopausal patient with Her2/neu positive breast cancer, who received the combined administration of bevacizumab and trastuzumab over a long period of time.

**FIGURE 1.**
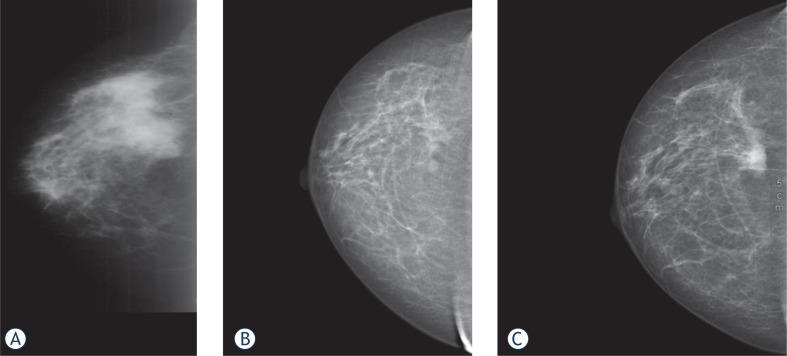
**A.** Low quality mammography showing a polycyclic lesion in the laterocranial quadrant with a diameter of 35 mm and a second lesion with a diameter of 20 mm in the centrocaudal quadrant. **B.** Mammography performed after 25 cycles of trastuzumab and bevacizumab. The former polycyclic lesion in the laterocranial quadrant now has a diameter of 4 mm. The second lesion in the cetntrocaudal quadrant is no more traceable. **C.** Mammography performed after 74 cycles of trastuzumab and bevacizumab. The lesion in the laterocranial quadrant progressed, measuring 20 mm in diameter.

A 58-year-old woman with a newly diagnosed cancer of the right breast was referred to our department for antineoplastic therapy. In order to better understand and justify our further management, the reader has to know that the patient had a history of a psychiatric disorder with long-standing delusional symptoms. She had discontinued antipsychotic drugs because of subjectively perceived worsening. Overall, the patient is socially well integrated. Other known co-morbidities were chronic impairment of renal function after nephrectomy following pyelo-nephritis and diabetes mellitus type II. The first low quality mammography was performed at the outpatient setting, showed two masses in the right breast, one lesion with a diameter of 35 mm and one of 20 mm (Figure 1A). During the diagnostic evaluation process, the patient refused to repeat the mammography. Biopsy revealed a multi-centric, invasive ductal, grade 2 carcinoma with lymphangiosis. Oestrogen receptor status (ERICA: SI 3, PP 4 (90%) IRS 12) and Her2/neu receptor status (DAKO lot 30586: 3+) were highly positive, progesterone receptor status was completely negative (PR-ICA: SI 0, PP 0, IRS 0), respectively. Thirty percent of tumor cells had a positive Ki-67 index.

The proposed classical preoperative cytotoxic chemotherapy with its potential side effects as well as mastectomy and axillary lymph node dissection were not reconcilable with the integrity of a female body image, and thus were categorically refused by the patient. Our therapeutic approach therefore focused on the immune-histochemistry data of the Her2/neu positivity and the use of new targeted, non-cytotoxic drugs. As a result, the patient was offered customized, albeit experimental treatment with the humanized monoclonal antibody trastuzumab (Herceptin^®^) combined with the humanized monoclonal antibody bevacizumab (Avastin^®^).

Therapy was initiated according to Table 1 and repeated on a three weekly base. For the first four cycles of combined antibody therapy the initial bevacizumab dosage of 10 mg/kg of body weight (BW) was chosen because the patient refused to accept the internationally recommended dosage of 15 mg/kg.

After the fourth cycle, a good partial response was documented by mammography. The second lesion with a diameter of 2 cm was and would be no more traceable throughout the forthcoming mammographies. After 25 cycles of double antibody therapy a further reduction of the tumor mass was observed (Figure 1B). As the patient did not cease refusing surgery categorically, the original treatment was consistently continued. After 8 months of treatment, the bevacizumab dosage was reduced to 7.5 mg/kg due to the patient′s request. After 51 cycles of combined antibody therapy the patient agreed to receive 15 mg/kg of bevacizumab, according to the recommendation for breast cancer treatment, because mammography presented a suspicious enlargement. The mammography performed after 48 months of therapy, confirmed the persistence of a partial remission compared to the initial outpatient mammography. Four years after diagnosis the patient was free of symptoms related to her malignant disease or the respective treatment which let us maintain therapy unchanged. However, after 74 cycles of combined antineoplastic therapy progression of the lesion was documented by mammography (Figure 1C).

Monitoring of potential cardiac abnormalities, including echocardiography and measuring of NT-proBNP levels, have been done repeatedly. Newly diagnosed hypertension was well controlled by ACE inhibitors.

## Discussion

VEGF is a well-established key-factor inducing angiogenesis leading to tumour growth and metastasis.^1^,^2^ There exists a significant correlation between tumour microvessel density in breast cancer, the presence of axillary lymph node and distant metastases, respectively.^3^ VEGF over-expression is frequently observed in Her2/*neu* positive patients with breast cancer.^4^,^5^ Via multiple intracellular pathways VEGF and Her2/*neu* act at various stages of breast cancer development.^6^ Over-expression of the proto-oncogene Her2/*neu* is associated with an up-regulation of VEGF *in vitro* and *in vivo*.^8^–^9^ Transfection of Her2/*neu* over-expression resulted in a rise of VEGF on RNA as well as on protein levels.^8^–^9^ *In vitro* VEGF was reduced by exposure to Her2/neu antibodies such as trastuzumab, especially in cells with Her2/neu over-expression.^7^,^9^,^10^ Considering VEGF as a possible downstream effector of Her2/*neu*, which might contribute to the more aggressive phenotype of Her2/neu over-expressing breast cancer cells, Konecny *et al.*, showed a significant association of Her2/neu over-expression and VEGF up-regulation based on tissue samples of 611 unselected breast cancer patients.^11^ In this study, VEGF expression was negatively correlated with survival. These results were concordant with the results of Linderholm *et al*., thus prompting to a re-evaluation of combined treatment strategies targeting both Her2/neu and VEGF.^12^ On the other hand a paper recently published by Liu *et al.* showed that in Her2/*neu* positive breast cancer patients VEGF over-expression was not significantly correlated with breast cancer-specific mortality, distant recurrence or overall mortality, respectively.^5^ These conflicting retrospective results regarding the possible prognostic and predictive value of VEGF over-expression are demanding prospective clinical studies evaluating the benefit of adding bevacizumab to trastuzumab in patients with Her2/neu positive breast cancer.

So far, in a clinical phase I trial, 9 patients were subjected to combination treatment with bevacizumab, 3.0, 5.0 or 10.0 mg/kg BW, respectively, at intervals of 14 days, and trastuzumab at a loading dose of 4 mg/kg BW, followed by 2 mg/kg BW once a week until progression.^13^ Grade 3 and 4 side effects were absent throughout. Grade 1 and 2 side effects consisted of diarrhoea, fatigue and nausea. In addition, one patient developed grade 2 allergic reactions, another one grade 2 hypertension and yet another one grade 2 proteinuria. Left ventricular function did not deteriorate. Bevacizumab combined with trastuzumab was well tolerated. After 6 cycles complete remission was recorded in one, partial remission in 4, stable disease in 2 and disease progression in 2 patients, respectively. Pharmacokinetic studies showed that the administration of the two drugs on the same day did not alter the pharmacokinetic patterns of either drug. According to this study, the dosage recommended for the phase II trials was 10 mg/kg BW every 14 days for bevacizumab and 4 mg/kg BW for loading followed by 2 mg/kg BW once a week for trastuzumab. In this study, one patient had progressed on prior chemotherapy and trastuzumab. Five of 9 patients improved clinically. These data argue in favour of combining anti-Her2/neu and anti-VEGF treatment in patients with Her2/neu-positive breast cancer.

In the very first phase II trial with a combination of these humanized antibodies in breast cancer^14^, the clinical efficacy of combination treatment with trastuzumab and bevacizumab as well as safety and toxicity were evaluated. Patients were initially given trastuzumab at a loading dose of 4 mg/kg BW and bevacizumab at a dose of 10 mg/kg BW on day 7. In the further course, trastuzumab was given at a dose of 2 mg/kg BW once weekly combined with bevacizumab, 20 mg/kg BW, at intervals of 2 weeks. Interim analysis of 37 patients treated accordingly showed complete remission in one patient, partial remission in 19 patients, stable disease in 11 and disease progression in 6 patients.

One multicenter phase III trial initiated by the NSABP (BETH Study) will determine the value of adding bevacizumab to chemotherapy plus trastuzumab in patients with resected node-positive or high risk node-negative, Her2/neu-positive breast cance.^15^

To make the regimen more convenient to our patient, we chose a three weekly cycle. This is justifiable nonetheless since bevacizumab displays linear pharmacokinetics, yielding similar exposure with flexible dosage regimens administered on a mg/kg basis such as bi- or three-weekly dosing.^16^ Pharmaco-dynamic information collected during clinical trials in phase I to III studies of bevacizumab showed that under treatment with bevacizumab at different dosages, *e.g*. at a dose of 2.5 mg/kg per week in colorectal cancer and at 5.0 mg/kg per week in breast cancer circulating VEGF levels were un-measurable.^17^,^18^

The initial intention to augment the dosage of bevacizumab to 15 mg/kg three weekly was finally reached because the patient could be convinced that the internationally recommended dosage of bevacizumab might suspend further tumor growth. Due to our patient′s request, she received initially 10 mg/kg bevacizumab. The dosage was reduced after 11 cycles to 7.5 mg/kg. But even with the lower dosage of bevacizumab further reduction of the tumor mass was observed. This observation might support the effectiveness of lower dosages of bevacizumab which is in line with previously published pharmaco-dynamic studies. On the other hand lowering the dosage of bevacizumab with no detrimental effect on the tumor size might indirectly indicate that the addition of bevacizumab to trastuzumab had little or no benefit which would be in line with some comparable phase III studies in metastatic breast cancer. However, the benefit of trastuzumab in Her2/neu positive breast cancer is indisputable. As a single agent in first-line treatment of Her2/neu positive metastatic breast cancer trastuzumab yielded objective response rate up to 26%.^19^ Another phase III study (ECOG 1105) currently evaluates this issue studying first-line chemotherapy and trastuzumab to compare how well they work when given with or without bevacizumab in treating patients with metastatic breast cancer that over-expresses Her2/neu.^20^

Currently, the combination of trastuzumab and bevacizumab in the first line treatment of HER2/neu positive breast cancer is not justified since there are other anti-HER2 drug combinations that have shown more striking results at least in the metastatic setting.^21^,^22^

## Conclusions

The attempt to act on several signalling pathways involved in tumor development simultaneously appears to be more attractive than attacking a single target structure alone. The combined double administration of bevacizumab and trastuzumab is easily handled, and represents a safe and non-toxic regimen allowing long-term application in patients with Her2/neu-positive recurrent, metastasizing as well as primary breast cancer. Targeting both Her2/neu and VEGF pathways was effective in our case for a long period of time although we can not say to what extent the benefit is attributed to the addition of bevacizumab and to what extent to trastuzumab solely.

## Figures and Tables

**TABLE 1.** Antineoplastic treatment plan of a patient with estrogen - and Her2/neu receptor positive breast cancer

**cycle**	**week**	**trastuzumab**	**bevacizumab**	**tumor size**
1	0	8 mg/kg	-	35 mm
2	3	6 mg/kg	10 mg/kg	
4	9	6 mg/kg	10 mg/kg	10 mm
...	...	...	...	
11	30	6 mg/kg	7.5 mg/kg	
…	…	…	…	
25	75	6 mg/kg	7.5 mg/kg	4 mm
…	…	…	…	
51	151	6 mg/kg	15 mg/kg	12 mm
…	…	…	…	
74	222	6 mg/kg	15 mg/kg	20 mm
